# Crosstalk between Vitamins A, B12, D, K, C, and E Status and Arterial Stiffness

**DOI:** 10.1155/2017/8784971

**Published:** 2017-01-12

**Authors:** Ioana Mozos, Dana Stoian, Constantin Tudor Luca

**Affiliations:** ^1^Department of Functional Sciences, Discipline of Pathophysiology, “Victor Babes” University of Medicine and Pharmacy, Timisoara, Romania; ^2^2nd Department of Internal Medicine, “Victor Babes” University of Medicine and Pharmacy, Timisoara, Romania; ^3^Department of Cardiology, “Victor Babes” University of Medicine and Pharmacy, Timisoara, Romania

## Abstract

Arterial stiffness is associated with cardiovascular risk, morbidity, and mortality. The present paper reviews the main vitamins related to arterial stiffness and enabling destiffening, their mechanisms of action, providing a brief description of the latest studies in the area, and their implications for primary cardiovascular prevention, clinical practice, and therapy. Despite inconsistent evidence for destiffening induced by vitamin supplementation in several randomized clinical trials, positive results were obtained in specific populations. The main mechanisms are related to antiatherogenic effects, improvement of endothelial function (vitamins A, C, D, and E) and metabolic profile (vitamins A, B12, C, D, and K), inhibition of the renin-angiotensin-aldosterone system (vitamin D), anti-inflammatory (vitamins A, D, E, and K) and antioxidant effects (vitamins A, C, and E), decrease of homocysteine level (vitamin B12), and reversing calcification of arteries (vitamin K). Vitamins A, B12, C, D, E, and K status is important in evaluating cardiovascular risk, and vitamin supplementation may be an effective, individualized, and inexpensive destiffening therapy.

## 1. Introduction

Cardiovascular diseases are the main cause of mortality worldwide and prophylactic measures deserve special attention. Arterial stiffness, one of the earliest detectable signs of structural and functional changes of the vessel wall [1], is associated with cardiovascular risk, morbidity and mortality, atherosclerosis and arteriosclerosis, aging, and several chronic disorders. Measurement of pulse wave velocity (PWV) is a simple, noninvasive, validated, the most used, and reproducible method to assess arterial stiffness [2]. A recently published meta-analysis, including 17,635 participants, demonstrated that an increase of PWV of 1 m/s is associated with a 7% increased risk of subsequent cardiovascular events, concluding that aortic PWV enables identification of high cardiovascular risk subjects, that might benefit from more aggressive risk factor management [3]. Augmentation index, a measure of peripheral arterial reflective properties, is also a complex and indirect marker of arterial stiffening [4, 5]. A 10% increase in the augmentation index was associated with 31.8% increased risk of cardiac events [6].

Aging and several disorders cause degenerative changes of the vessel wall of large arteries, related to the rupture of the elastic fibers, impaired cross-linking of extracellular matrix components, accumulation of collagen, fibrosis and necrosis of muscle fibers, inflammation, and calcification, leading to their stiffening [7, 8]. Vascular calcification, calcium phosphate complexes deposition in the arterial wall, is an active process, enabled by several mechanisms, and leads also to loss of arterial wall elasticity and an increased PWV, related to vascular remodeling, organ damage, and overall morbidity and moratlity [9, 10]. It can be present as medial calcification (Monckeberg*ʼ*s medial sclerosis, prevalent among patients with diabetes and renal and hyperparathyroid disorders) or intimal calcification (on the surface of the atherosclerotic plaque) [11]. Hypertension, inflammation, oxidized low density lipoproteins, and a high calcium-phosphorus ion product enable transformation of vascular smooth muscle cells into osteocyte-like cells [12, 13]. Functional deterioration of the arteries involves reduced bioavailability of NO and endothelial dysfunction [1]. Reduction of arterial compliance causes a faster reflection of the systolic wave from the peripheral arteries to the heart, increasing the central aortic pressure and causing myocardial hypertrophy and ischemia [1].

Destiffening is a challenge of cardiovascular prevention and deserves special attention. Diet is a modifiable cardiovascular risk factor [14] and Mediterranean diet and nutrients with antioxidant and anti-inflammatory properties may improve vascular function, despite contradictory findings of randomized trials [15–17].

There is a worldwide trend toward nutritional insufficiency [18], related to aging of the population, with changes of anatomy and function of the kidney. Socioeconomic improvement has been associated with nutritional changes and increased prevalence of cardiometabolic disorders [19]. Dietary factors may accelerate or slow the evolution of cardiovascular disorders. Dietary vitamin supplements, easily accepted and often used by Western populations, may provide potential benefits and harms related to their use [20].

Considering the worldwide trend toward nutritional insufficiency, it was the aim of the present paper to review the main vitamins related to arterial stiffness and enabling destiffening, their mechanisms of action, including a brief description of the latest studies in the area, and their implications for primary cardiovascular prevention, clinical practice, and therapy.

## 2. Vitamin D

Vitamin D, “the sunshine vitamin,” is known especially for promoting calcium deposition in bones. Vitamin D receptors have been found in several other tissues, including vascular smooth muscle, endothelial cells, and cardiomyocytes [27]. Vitamin D deficiency is very common, due to indoor lifestyle, sun avoidance strategies, air pollution, and smoking, is often unrecognized or untreated, and is associated with increased all-cause mortality and cardiovascular event rate [28] and linked to major cardiometabolic risk factors, including obesity, hypertension, and diabetes mellitus [50]. Unfortunately, it is also very common during perinatal period, linked not just with poor bone development, but also with heart disease, type 1 diabetes mellitus, and cancer [51, 52]. Vitamin D deficiency* activates the renin-angiotensin-aldosterone system*, and the increased vascular tone, due to release of angiotensin II, and arterial stiffness precede the development of hypertension [53]. Vitamin D has also pleiotropic effects on the* immune system* and* suppresses the low grade inflammation in the cardiovascular system*, downregulating Th1 activity and dendritic cell maturation, inhibiting production of cytokines, and upregulating Th17 regulatory activity and modulating macrophage activity [28, 54, 55].

Usually* 25 hydroxy vitamin D* and not 1,25 dihyrdoxy vitamin D is assessed. 25 hydroxy vitamin D is the nutritional parameter of vitamin D status, the primary circulating and storage form of vitamin D in the human body, a reliable, available marker of low vitamin D levels, easy to administer, with few side effects, and able to bind to vitamin D receptors [5, 28]. In the Whitehall study, the optimal concentration of 25(OH) vitamin D was 80–90 mmol/l and a linear, inverse association of vitamin D level with both vascular and nonvascular mortality was reported [56].

The role of vitamin D deficiency in vascular disease is an emerging issue [18]. Several studies revealed associations between vitamin D level and PWV [5, 57–60], but other authors found no significant association between vitamin D concentrations and markers of subclinical atherosclerosis [61, 62]. The effect of vitamin D supplementation on arterial stiffness showed also conflicting results, either beneficial or not (Table 1). Daily 2,000 IU vitamin D supplementation for 16 weeks resulted in a significant decrease in carotid-femoral PWV in 25 normotensive black boys and girls [33]. A significant decrease of arterial stiffness was also noticed after a single dose of 300,000 IU of cholecalciferol in children with chronic kidney disease [22]. Zaleski et al. reported a decrease of arterial stiffness with high-dose vitamin D supplementation for 6 months, and no effect on blood pressure [24]. The pleiotropic beneficial effects of vitamin D on arterial stiffness were suggested to be* dose-dependent*, which could explain the conflicting results of different studies [24]. McGreevy et al. found a significant decrease in augmentation index after 100,000 IU of vitamin D3 (the high-dose group) for 8 weeks, in older adults with vitamin D deficiency [25]. Forouhi et al. showed a modest reduction in PWV after D2 and D3 supplementation [21]. Ryu et al. found no beneficial effect of high-dose vitamin D supplementation on cardiovascular risk factors, PWV, or augmentation index in diabetic patients [27] and the same lack of change was reported by Chitalia et al. in predialysis chronic kidney disease patients [28]. Six months of vitamin D supplementation did not decrease central blood pressure parameters or arterial stiffness in healthy postmenopausal native American women [26]. A recent meta-analysis of randomized controlled trials concluded inconsistent evidence for destiffening induced by vitamin D supplementation, attributable to the heterogenity of the included studies [63].

*Vascular calcification* in the coronary or peripheral arteries is a powerful predictor of cardiovascular morbidity and mortality, linked to PWV [10], especially in hemodialysis patients [64]. High concentrations of vitamin D enable increased gastrointestinal calcium absorption, resulting in higher circulating calcium concentrations and increasing the number and size of calcification foci, resulting in vascular calcification, especially in atherosclerotic plaques [9, 62, 65]. Sachs et al. observed associations of lower concentration of vitamin D metabolites with reduced coronary artery calcium prevalence and severity, measured by computed tomography, in patients with type 1 diabetes mellitus [61]. A mouse model of chronic kidney disease revealed protective effect against aortic calcification at low vitamin D3 doses, sufficient to correct secondary hyperparathyroidism (higher doses stimulated aortic calcification) [66].

The main pathophysiological vasculoprotective mechanisms by which vitamin D supplementation reduces arterial stiffness include decrease of the renin-angiotensin-aldosterone system activity, suppression of endothelin-induced vascular smooth muscle cell proliferation, renoprotective effects, effects on calcium metabolism and PTH level, counterbalance of inflammation and oxidative stress, and improvement of carbohydrate metabolism and insulin sensitivity [24, 29, 33, 67, 68]. Fibroblast growth factor 23 (FGF-23), a circulating peptid secreted by bone cells, known for inducing phosphaturia, lowering 1,25 dihydroxy-vitamin D, and suppressing PTH secretion, a new marker of inflammation, insulin resistance, and visceral fat accumulation, has been also associated with cardiovascular events and arterial stiffness [50, 69]. FGF-23 acts on the vascular function through its coreceptor Klotho, which increases nitric oxide availability and it has also been related to the presence of vascular calcifications [50, 70]. Low levels of vitamin D were associated with increased activity of matrix metalloproteinases and C reactive protein, correctable by supplementation [71].

The inconsistencies of vitamin D supplementation trials could be attributable to heterogeneity in vitamin D dosage, compounds and baseline concentration, study duration, design, population and follow-up, lack of a control group with normal vitamin D level, biases due to different comorbidities, therapy known to affect arterial stiffness, and several other confounding factors [24, 72]. Further large, longer duration, controlled randomized studies are required in order to demonstrate a causal relationship between vitamin D supplementation and decrease of arterial stiffness and to understand the importance of maintaining vitamin D sufficiency. There is no consensus on optimal levels of vitamin D in order to provide a beneficial cardiovascular effect, and this will be the aim of future clinical trials.

## 3. Vitamin K

Vitamin K, a lipid-soluble vitamin, is an essential micronutrient. It includes vitamins K1, K2, and K3. Phylloquinone (vitamin K1, phytonadione) is found especially in dark-green leafy vegetables and seeds, and menaquinone-7 (Vitamin K2), formed mostly by bacteria, may be obtained from meat, eggs, and fermented cheese [11, 73]. Vitamin K3 (menadione) is a synthetic vitamin K.

Vitamin K is essential for blood coagulation, but vitamin K insufficiency was associated also with an increased risk of cardiovascular events in healthy middle-aged people, type 2 diabetes, and end-stage renal disease patients [34]. Observational studies revealed a lower prevalence of arterial calcification and cardiovascular mortality in subjects with a high intake of menaquinones (vitamin K2) and no effect for phylloquinone (vitamin K1) [35, 74, 75]. An accelerated progression of aortic stiffness, associated with the use of warfarin, has been demonstrated in a study including 18 hemodialysis patients [76]. Vitamin K antagonists inhibit the recycling of vitamin K in the epoxide cycle, reduce carboxylation of coagulation factors, and cause calcifications in several arteries and valves [11]. Progression of aortic stiffness was related to the severity of vitamin K deficiency, assessed using circulating PIVKA-II, the undercarboxylated level of prothrombin and a sensitive subclinical vitamin K deficiency marker [76]. Matrix Gla-protein (MGP) is an inhibitor of soft tissue calcification; in its mature, active form it contains five Gla-residues (resulting from vitamin K-dependent gamma-carboxylation of the respective Glu-residues) and three phosphoserine residues [11, 76, 77]. The inactive form, desphospho-uncarboxylated MGP (dp-ucMGP) is regarded as a sensitive marker for vascular vitamin K status and increased levels of plasma dp-ucMGP are associated with increased cardiovascular and all-cause mortality [11, 35, 78]. As calcification develops, MGP is upregulated in vascular smooth muscle cells, as a negative feed-back mechanism [11, 79]. Pivin et al. found a positive association between PWV and inactive MGP, before and after adjustment for common cardiovascular risk factors and renal function [80]. Mayer Jr. et al. confirmed the relationship between inactive MGP and aortic stiffness, after adjusting for all potential confounders, but not with stiffness of muscular-type arteries [81]. MGP levels were not predictors for carotid-femoral pulse wave velocity in renal transplant recipients [82].* Vitamin K-dependent proteins, *requiring carboxylation to become biologically active, contribute to thrombus formation, vascular calcification, vascular stiffness, and ischemic cardiovascular events [11, 83].

A possible role of vitamin K in* suppressing chronic inflammation* and* reversing calcification of arteries* was suggested [34]. Observational studies have shown an inverse association between vitamin K status and inflammatory markers, such as interleukin-6 and C reactive protein [35, 84]. Vitamin K might suppress inflammation by decreasing expression of genes for cytokines [85]. Warfarin may impair pulse pressure in patients with a history of hypertension and higher cardiovascular risk [86], contributing to progression of arterial stiffness. Vitamin K2 has also the ability to* improve lipid profile* by increasing HDL cholesterol and decreasing total cholesterol [74].

Induction of type 1 diabetes mellitus, in rats with streptozotocin, resulted in augmentation of arterial stiffness, increase of aortic and femoral calcifications, and reduction of *γ*-carboxylated MGP (the active form of MGP) [8]. Reduced MGP is involved in the early development of medial artery calcification (MAC) in diabetes and the deposition of hydroxyapatite crystals along the large arteries, resulting in accelerated arterial stiffness [8].

A correlation between PWV and aortic calcium content has been previously demonstrated in a warfarin-vitamin K rat model of MAC [7]. Increased vitamin K2 intake has been associated with decreased arterial calcium deposition and the ability to reverse vascular calcification in animal models [10]. Vaccaro and Huffman found an inadequate vitamin K1 intake in older adults, especially in Hispanic and Black Americans, and vitamin K1 was an independent predictor of high arterial pulse pressure [87].

Daily supplementation with oral vitamin K2 for 6 months caused a modest, nonsignificant improvement in PWV in older patients with vascular disease [34] (Table 2). Long-term supplementation with 180 *μ*g menaquinone/day for three years decreased arterial stiffness in healthy postmenopausal women, especially in women with a high arterial stiffness [35]. Spronk et al. fed warfarin-treated rats diets containing vitamins K1 or K2 and found that just menaquinone inhibits warfarin-induced arterial calcification, explained by its more effective utilization in the aorta [88]. The VitaK-CAC trial will explore the effect of menaquinone-7 supplementation on progression of coronary artery calcification in a randomized trial [36].

Concluding, vitamin K, especially K2, enables destiffening by impairing and reversing calcification of arteries, suppressing the inflammatory reaction in the vascular wall and improving the lipid profile. Additional intake of vitamin K2 does not increase procoagulant activity or thrombosis risk because the blood coagulation factors are fully carboxylated [35].

## 4. Vitamin E

Vitamin E, the most abundant fat soluble* antioxidant* in the human organism, is found in high concentrations in palm oil, rice ban, and oily plants [41, 89] and includes tocopherols and tocotrienols. Plasma concentrations of vitamin E depend on the amount of plasma lipids and LDL cholesterol, considering that the latter is the main plasma carrier of tocopherol [90]. There is no generally accepted recommendation for an adequate intake of vitamin E [89]. Concentrations of vitamin E are influenced by age, lifestyle factors, such as obesity, smoking, alcohol consumption, fat malabsorption, interindividual differences in vitamin E metabolism, and interaction with pharmaceuticals (vitamin K, statins) [89].

Dietary intake of alpha-tocopherol, the main component of vitamin E, may reduce the cardiovascular risk [91, 92]. The antiatherogenic role of vitamin E has been suggested by its ability to* decrease LDL oxidation* [91, 93],* quench free radicals*,* inhibit protein kinase C* (PKC),* inhibit expression of adhesion molecules* and monocyte transmigration [47, 94], and* impair vascular smooth muscle cell proliferation* [93]. PKC is a key mediator of the vasoconstrictor response to oxidative stress [95], and the inhibition of PKC is another mechanism enabling improvement of vascular endothelial function besides the antioxidant action [47]. Vitamin E may become* prooxidant* at high doses, in the absence of an effective cooxidant, enabling production of alpha-tocopheroxyl radical, which can be inhibited by coantioxidants such as vitamin C [46, 96, 97]. Vitamin C reverses the prooxidant state of vitamin E, supporting the combined administration of vitamins C and E [98, 99]. But vitamin C can become prooxidant as well [98]. Human studies did not confirm a reduction of oxidative stress biomarkers due to combined administration of vitamins E and C, and the synergistic effects of the two vitamins disappeared under anaerobic conditions and became prooxidant [99]. Vitamin E has also* anticoagulant* properties by interfering with vitamin K-dependent clotting mechanisms not related to its antioxidant effect [89].

Conflicting results have been obtained regarding effectiveness of vitamin E in reducing atherosclerosis progression or lipid lowering effect [41]. Vitamins E and C* increased the collagen content of the arterial wall* and reduced vascular metalloproteinase-1, stabilizing the atherosclerotic plaque in a porcine model of atherosclerosis [100]. Antioxidant vitamins inactivate free radicals, increase plasma and tissue antioxidant defense,* reduce inflammation*, inhibit DNA oxidation by H_2_O_2_ in human lymphocytes, and* restore endothelial function* [16, 101, 102].

Hampson et al. found significant associations between PWV and alpha-tocopherol/gamma-tocopherol ratio, but not with alpha-tocopherol and gamma-tocopherol, in a cross-sectional study, including 278 postmenopausal women, in a multilinear regression model, adjusted for lipids, age, and blood pressure, emphasizing the importance of the balance between the two vitamin E isomers in maintaining arterial compliance [103].

Short term supplementation with 1,000 IU of vitamin E improved endothelial function but not systemic arterial compliance in young patients with type 1 diabetes mellitus [47]. The improvement in endothelial vascular function was related to a reduction of LDL oxidation [47], and there is evidence that oxidized LDL might inactivate endothelial cell derived relaxing factor [104]. The endothelium, through the generation of vasoactive mediators (NO and endothelin), might influence arterial stiffness [105]. Wigg et al. demonstrated improved endothelium-dependent and independent vasodilation in mesenteric arteries from diabetic rats, associated with PKC, after vitamin E, independent of advanced glycation end-product accumulation [106]. In the femoral artery, vitamin E prevented the wall stiffening associated with diabetes [106]. The benefit may be due to a direct effect of vitamin E on smooth muscle function as a consequence of inhibition of PKC-*β*2 isoform and due to improved NO availability in the smooth muscle [106]. Vitamin E supplementation significantly improved the endothelial function, despite heterogeneity of the studies, especially in study participants with a lower baseline plasma vitamin E concentration (less than 20 *μ*M), and did not depend on age, vitamin E dose, and duration of supplementation [99].

Rasool et al. used low, medium, and high doses of vitamin E as self-emulsifying tocotrienol rich vitamin E, for 2 months, in 36 healthy men and reported a significant reduction of PWV and augmentation index for the groups 100 and 200 mg, and no change in blood pressure, serum total, and LDL cholesterol [41]. A systematic review and meta-analysis revealed a small protective effect of antioxidant vitamins (vitamins C, E, A, and beta-carotene) on arterial stiffness, especially in younger healthy participants compared to those with cardiometabolic disease, more important in subjects with lower baseline plasma level of vitamins C and E [102]. The beneficial effects of the combined treatment with antioxidant vitamins C and E are due to the reduction of the damaging effects of free radicals on the vascular components and the anti-inflammatory effect, increasing the bioavailability of NO, improving endothelium-dependent vasodilation, and decreasing arterial stiffness and protecting the integrity of the vascular wall [43, 102]. The rate constant of the reaction of antioxidant vitamins and superoxide is lower than that of the reaction between NO and superoxide, which makes the antioxidant vitamins ineffective in protecting NO from free radical inactivation [99]. Additionally, systemic chronic inflammation may accelerate aging due to reactive oxygen species-mediated exacerbation of telomere dysfunction and cell senescence [107]. Natural antioxidants such as vitamins C and E, the beneficial components of fruits and vegetables, may exert toxic prooxidant activities at higher doses or under certain conditions [98], vitamin E being able to depress myocardial function [108]. Alpha-tocopherol may suppress other fat soluble, more powerful antioxidants such as gamma-tocopherol, increasing vulnerability to oxidative damage [109]. Miller III et al. concluded, in a meta-analysis, that there is a* dose-dependent* relationship between vitamin E supplementation and all-cause mortality and that high doses of vitamin E (≥400 IU) may increase all-cause mortality and should be avoided [97]. All-cause mortality progressively increased for doses exceeding 150 IU/day, substantially lower than the tolerable upper intake level for vitamin E (1,500 IU natural or 1,100 IU of synthetic vitamin E) [97]. No relation was observed between brachial-ankle PWV and alpha-tocopherol in a study including 178 Japanese male workers [110].

Shargorodsky et al. demonstrated that combined antioxidant supplementation with vitamins C and E, coenzyme Q10, and selenium improves glucose and lipid metabolism, blood pressure, and arterial compliance in patients with at least 2 cardiovascular risk factors [16].

In the study of Veringa et al., 93 patients with chronic kidney disease received pravastatin, vitamin E, and homocysteine lowering therapy, resulting in significant improvement of arterial compliance and distensibility, but the effect of vitamin E is not clear because it was combined with pravastatin [40].

Vucinovic et al. demonstrated that the acute intake of an antioxidant cocktail, including vitamins C and E, preserved bioavailability of NO and vascular function against hyperoxia-induced oxidative stress [37] (Table 3). Combined supplementation of vitamins C and E was ineffective in improving endothelial function in 14 randomized trials, including 597 participants, regardless of age, duration, dose, or baseline plasma concentration of vitamins [99]. Park et al. demonstrated that higher intake of beta-carotene, vitamins C, E, and folate may protect individuals genetically vulnerable to stiffening of the arteries, in a study including 3,198 healthy men and women from the Korea Multirural communities, quantifiyng dietary intakes by a food frequency questionnaire [111].

Concluding, vitamin E supplementation is supposed to decrease arterial stiffness due to its antiatherogenic and antioxidant effects and the ability to restore endothelial function, but its prooxidant effect must be considered at higher doses. Meta-analyses failed to confirm the role of antioxidant supplementation in primary and secondary cardiovascular prevention and recommend combined use of antioxidants [112]. Several limitations have been noticed in studies evaluating the effect of antioxidant vitamins on arterial stiffness and cardiovascular risk, such as a small sample size, heterogenous study populations, lack of objective criteria to include participants as healthy, differences in definition criteria of patients who are potential candidates for antioxidant therapy, different type and dosage of antioxidants, low correlations between dietary vitamins C and E intake, and plasma levels, reflecting the innacuracy of dietary questionnaires in the assessment of intake, individual variations in vitamin absorption and metabolism, missing plasma levels of antioxidants, and data regarding treatment compliance [16, 102, 108]. When excluding small studies (with less than 20 participants), antioxidant vitamin supplementation in larger studies significantly decreased arterial stiffness [102].

## 5. Vitamin C

Ascorbic acid is the cofactor of hydroxyproline synthesis, stabilizing the triple helix structure of* collagen.* Vitamin C is also a potent water-soluble* antioxidant*, enabling scavenging of superoxide anions and other reactive oxygen species [38], and prevents LDL oxidation, through recycling of alpha-tocopherol or by directly scavenging free radicals [46]. Arterial stiffness is impaired by oxidative stress and negative correlations were obtained between PWV and superoxide dismutase level [113]. PWV was dependent on the free radical/antioxidant Redox balance and nitric oxide bioavailability in patients with chronic obstructive pulmonary disease, and PWV increased after an antioxidant cocktail including vitamins C and E and alpha-lipoic acid [39] (Table 3). Results of antioxidant using trials, as an intervention in cardiovascular disease, have been mixed, but it is important to mention that most trials used a single antioxidant. Kelly et al. reported no effect of a single dose of oral vitamin C on augmentation index and several markers of oxidative stress, including DNA base oxidation products, in 26 healthy volunteers [42]. On the other hand, Katayama et al. suggested that oral vitamin C administration prevents smoking-induced acceleration in arterial stiffness through* reducing endothelial dysfunction*, but does not influence heart rate and blood pressure [44]. The long-term effect in smokers is not beneficial on endothelial function [114] and smoking negatively influenced vitamin C level [115]. The use of an antioxidant cocktail, containing both water and fat soluble vitamins, seems to be more beneficial for vascular stiffness [39]. Acute administration of vitamin C has also been previously reported to lower augmentation index in healthy volunteers [116].

Peripheral and central hemodynamic changes noticed in acute systemic hyperglycemia, may be prevented or attenuated by pretreatment with a 2 g intravenous bolus of ascorbic acid according to a study including 12 healthy men [45]. Ascorbic acid may reverse impaired endothelium-dependent NO-mediated vasodilation in several conditions, including acute hyperglycemia and chronic renal failure [45, 117]. The main mechanisms of* NO increase* include reduced NO degradation by free radicals considering ascorbic acid, as an extremely potent free radical scavenger, increase of endothelial NO synthase activity, increase in the intracellular content of tetrahydrobiopterin, reduced insulin resistance, or smooth muscle sensitivity to NO [45, 46, 116, 118, 119]. Diabetes mellitus is associated with endothelial dysfunction due to several factors, including hyperglycemia, insulin resistance, hyperlipidemia, oxidized LDL and ascorbic acid deficiency (due to an impaired vitamin C recycling), and arterial stiffness [46]. Oral administration of 500 mg ascorbic acid/day, for 4 weeks, reduced arterial stiffness in diabetic patients, suggesting a functional change, probably due to increased NO [46].

Vitamins C and E increased the collagen content of the arterial wall and reduced vascular metalloproteinase-1, which explains their role in the* structural remodeling of the vessel wall* and stabilizing the atherosclerotic plaque [100].

Stiffening of large arteries increases progressively during menopause, mediated by estrogen deficiency, oxidative stress, and reduction of NO bioavailability [38]. Infusion of supraphysiological doses of ascorbic acid increased carotid artery compliance in late perimenopausal and postmenopausal women, but not in premenopausal women; the carotid artery compliance was not restored to premenopausal levels [38]. Late perimenopause is associated with changes in lipid metabolism and cardiovascular risk factors, known amplifiers of oxidative stress and arterial stiffening [38, 120]. Incomplete suppression of reactive oxygen species (ROS) by ascorbic acid and involvement of other sources of ROS, such as peroxynitrite, could explain why artery compliance was not restored to premenopausal levels [38].

High doses of vitamin C may have a* prooxidant* effect [98] and may impair arterial stiffness. On the other hand, only supraphysiological concentrations of ascorbate may prevent the interaction of superoxide and nitric oxide [46, 121]. Higher vitamin C levels were associated with lower levels of* inflammatory markers*, fasting blood glucose, and improved endothelial function [108, 122]. Supplementation with vitamin C alone improved endothelial function, especially in study participants older than 56 years; no significant modifying effects of the dose or duration of vitamin C supplementation on endothelial function were found [99]. Older people are more likely to have inadequate micronutrient intakes and absorption and greater oxidative stress due to age-related mitochondrial dysfunction, with a greater benefit from vitamin C supplementation [99].

Concluding, vitamin C supplementation may reduce arterial stiffness by stabilizing the atherosclerotic plaque and its antoxidant and anti-inflammatory effect and by improving endothelial function.

## 6. Vitamin A

Vitamin A includes several organic compounds, such as retinol, retinoic acid, and carotenoids (lycopene, lutein/zeaxanthin, beta-carotene, alpha-carotene, and beta-cryptoxanthin). Besides vitamin A importance in good vision and immune system, it is included in the group of* antioxidant *vitamins [102]. High serum concentrations of carotenoids, abundant in many fruits and vegetables, associated with the Mediterranean diet, may be protective against early atherosclerosis, by* inhibiting LDL oxidation* [123, 124]. High serum concentrations of lycopene and alpha- and beta-carotene were associated with reduced intima-media thickness progression during 7-year follow-up. Vessel walls of carotid arteries were more elastic in subjects whose diets were rich in carotenoids according to the ARIC study [125]. Rissanen et al. found also low plasma lycopene levels associated with early atherosclerosis, manifested as increased intima-media thickness of the common carotid artery wall, in 520 middle-aged men and women living in eastern Finland [126].

Due to the antioxidant activity that attenuates the inflammatory atherosclerotic process, the carotenoids* delay vascular aging* due to several mechanisms: their antioxidant activity (that attenuates the inflammatory atherosclerotic process), the ability to* increase bioavailability of NO*, the* improvement of the metabolic profile*, and their LDL lowering effect [127].

Free radicals, resulting from smoking, deplete serum carotenoid levels, especially alpha- and beta-carotene, lutein/zeaxanthin, and beta-cryptoxanthin [115]. Vitamin A supplementation may slow progression of atherosclerosis, by reducing the production of the* inflammatory cytokine* IL-17 and retinoid-related orphan receptor-c gene expression, the main transcriptor factor that controls Th17 cells differentiation [128].

In other words, vitamin A supplementation enables destiffening due to its antioxidant and anti-inflammatory effect and by improving endothelial function and metabolic profile. Carotenoid utilization failed to decrease the rate of major cardiovascular events in randomized trials, and their role in secondary cardiovascular prevention is not clear [127], but further follow-up studies are needed in order to confirm its importance.

## 7. Vitamin B12

Vitamin B12 (cobalamin) is an essential, water-soluble nutrient, involved in DNA synthesis [19]. Vitamin B12 deficiency, very prevalent in Europe, is caused mainly by vegetarianism and is known to be associated with megaloblastic anemia and neuropathy. Metformin therapy, a first line therapy in type 2 diabetes mellitus, reduces the circulating B12 levels by 25% [129].

Several studies investigated the effect of vitamin B12 level and supplementation and cardiovascular health and arterial stiffness. Previous results suggested also associations between vitamin B12 level and* adverse serum lipid profiles* in patients with type 2 diabetes mellitus, especially with triglycerides and cholesterol/HDL ratio, due to inhibition of carnitine palmitoyl transferase, the rate-limiting enzyme of fatty acid oxidation [129]. Vitamin B12 deficiency caused also* elevation of homocysteine*, a risk factor for cardiovascular disease, by inhibiting its conversion to methionine [19]. It has been hypothesized that low B12 vitamin increases cardiovascular risk, partly through direct effects [130].

Su et al. found no significant differences of arterial function parameters between postmenopausal vegetarians and omnivores [131]. Just brachial artery resistance was lower in vegetarians [131].

Van Dijk et al. did not report any effect of vitamin B12 and folic acid supplementation on PWV or carotid intima-media thickness in hyperhomocysteinemic elderly patients [48] (Table 4).

Koyama et al. found decreased arterial stiffness, associated with decreased serum asymmetric dimethylarginine in 20 patients undergoing hemodialysis after supplementation with folate and methylcobalamin [49]. Vitamin B12 levels were marginally associated with PWV in 86 patients with diabetes mellitus [132].

Vitamin B12 supplementation enables destiffening by improving the lipid profile and reducing the homocysteine level. A systematic review of cohort studies concluded that current data do not support vitamin B12 supplementation to reduce cardiovascular risk [130]. Further long-term follow-up studies should focus on specific populations in order to confirm destiffening through vitamin B12 supplementation, not just in patients undergoing hemodialysis.

## 8. Conclusions

There is a complex relationship between vitamin status and arterial stiffness, and each vitamin has specific effects on the vascular wall. Vitamin supplementation may be an effective and inexpensive adjunctive therapy in several conditions associated with increased arterial stiffness and they should be implemmented in patients' diet, considering individual vitamin status.

Vitamin D deficiency, involved in the pathophysiology of cardiovascular disease, may be an important therapeutic target. Despite heterogeneity and conflicting results of trials on vitamin D supplementation, arterial stiffness was significantly decreased in children with chronic kidney disease, black adolescents, adults with vitamin D deficiency with or without prehypertension, nondiabetic patients with hyperglycemia or positive diabetes score, and type 2 diabetic patients with nephropathy. Further large, randomized, evidence based, follow-up studies, including subjects with several other disorders, will demonstrate if vitamin D level is a marker of subclinical atherosclerosis, an effective target in cardiovascular prevention, therapy, destiffening, and vascular protection, or just a marker of poor health status, and which is the most effective form and level of vitamin D.

Vitamin K was beneficial in decreasing arterial stiffness in healthy postmenopausal women, patients with a history of vascular or coronary artery disease, vitamin E in subjects with type 1 diabetes mellitus and chronic kidney disease, and vitamin C in smokers, late perimenopausal, and postmenopausal women, and patients with type 2 diabetes mellitus. The combination including vitamin C and E could play an important role in cardiovascular disease prevention in young participants with lower baseline plasma levels, resulting in decreased arterial stiffness in patients with chronic obstructive pulmonary disease and essential hypertension. Further studies are needed in order to explore the effect of vitamin A supplementation on arterial stiffness, considering the antioxidant effect of vitamin A, its effect on endothelial function, metabolic profile, and its anti-inflammatory effect. Vitamin B12 supplementation was demonstrated to reduce arterial stiffness in patients undergoing hemodialysis.

The divergent results and mismatch between epidemiological and interventional studies warrant further investigation, but vitamins A, B12, D, K, C, and E may be markers of arterial stiffness and cardiovascular health. Cardiovascular prevention guidelines should consider and include trials with positive results. Vitamin K2 and low dose vitamin D have promising potential for prevention of vascular calcification. The potential public health importance of vitamin level and supplementation remains to be further tested in stratified intervention studies, and future research should focus on optimal vitamin levels and identifying patients who would benefit most from vitamin supplementation in order to enable individualized therapy, a personalised approach, and early interventions in primary, but also secondary prevention of cardiovascular disease.

## Figures and Tables

**Table 1:** Vitamin D supplementation and arterial stiffness studies.

Authors	Year of publication	Vitamin D dose	Follow- up	Participants	Results
Forouhi et al. [21]	2016	100,000 IU/month vitamin D2 or D3	4 months	340 nondiabetic patients with hyperglycemia or positive diabetes risk score	Modest reduction in PWV

Aytaç et al. [22]	2016	Single dose of 300,000 oral cholecalciferol	12 weeks	41 children with chronic kidney disease	Significant lower arterial stiffness

Munisamy et al. [23]	2016	0.25 *μ*g alfacalcidol/day	6 months	28 type 2 diabetic nephropathy patients	Decrease of arterial stiffness

Zaleski et al. [24]	2015	4,000 IU/day versus 400 IU/day	6 months	40 vitamin D deficient adults with prehypertension	High dose of vitamin D lower arterial stiffness

McGreevy et al. [25]	2015	50,000 IU or 100,000 IU D3	8 weeks	119 vitamin D deficient subjects	Significant decrease in augmentation index in the high dose group

Gepner et al. [26]	2015	400 IU or 2,500 IU vitamin D	6 months	98 healthy postmenopausal native American women	No decrease of arterial stiffness

Ryu et al. [27]	2014	Cholecalciferol 2.000 IU/day	24 weeks	40 type 2 diabetes	No beneficial effect on arterial stiffness, cardiovascular risk, insulin resistance

Chitalia et al. [28]	2014	300,000 IU cholecalciferol at baseline and 8 weeks	16 weeks	26 nondiabetic patients with chronic kidney disease	Improvement of endothelial function, no change of arterial stiffness

Martins et al. [29]	2014	100,000 IU monthly	3 months	130 overweight and obese African Americans with elevated blood pressure	Decreased level of inflammatory and oxidative stress mediators of arterial stiffness, but not a decrease of arterial stiffness

Levin et al. [30]	2014	5,000 IU 25 vitamin D and 0.5 *μ*g 1,25 vitamin D 3 times/week in oral suspension for 6 months	9 months	128 stable chronic kidney disease patients	No results published yet

Mose et al. [31]	2014	3,000 IU cholecalciferol/day	6 months	50 chronic dialysis patients	No decrease of 24-hour blood pressure, arterial stiffness or cardiac function

Klop et al. [32]	2014	100,000 IU vitamin D3	Single dose	6 men and 6 women	Reduction of augmentation index, reduced postprandial leukocyte activation

Stricker et al. [4]	2012	100,000 IU vitamin D3	Single dose	62 elderly patients with peripheral arterial disease and low vitamin D levels	No influence on endothelial function, arterial stiffness, coagulation and inflammation parameters

Dong et al. [33]	2010	2,000 IU/day	16 weeks	25 Normotensive black boys and girls	Significant decrease in carotid-femoral PWV

**Table 2:** Effect of vitamin K supplementation and arterial stiffness in human subjects.

Authors	Year of publication	Follow- up	Dose of vitamin K	Study population	Results
Fulton et al. [34]	2016	6 months	Oral 100 mcg vitamin K2	Participants aged ≤70 years, with a history of vascular disease	A modest nonsignificant decrease in pulse wave velocity (PWV)

Knapen et al. [35]	2015	3 years	180 *μ*g menaquinone	120 healthy postmenopausal women	carotid-femoral PWV and the stiffness index *β* decreased

Vossen et al. [36]	2015	24 months	360 microgram menaquinone-7 (MK-7)	Patients with coronary artery disease	Difference in coronary artery calcification score between MK-7 and control group

**Table 3:** Effect of vitamin E and C supplementation and arterial stiffness in human subjects.

Authors	Year of publication	Follow-up	Dose of vitamin E and C	Study population	Results
Vucinovic et al. [37]	2015	1 week	600 IU vitamin E	12 healthy males	Hyperoxia resulted in increased augmentation index and lipid peroxides and decreased nitrite in placebo; not in the antioxidant group
			1,000 mg vitamin C		
			600 mg alpha-lipoic acid		

Hildreth et al. [38]	2014	—	Infusion of 7.5 g ascorbic acid	97 healthy women (premenopausal, perimenopausal and postmenopausal)	Improvement of arterial compliance in late perimenopausal and postmenopausal women

Ives et al. [39]	2014	90 minutes	Oral antioxidant cocktail	30 patients with chronic obstructive pulmonary disease	Vascular dysfunction mediated by an altered redox balance can be mitigated by an oral antioxidant; the antioxidant cocktail improved also PWV
			(1) Dose: 300 mg alpha lipoic acid, 500 mg vitamin C, 200 IU vitamin E		
			(2) Dose: the same doses of alpha lipoic acid and vitamin C, 400 IU vitamin E		

Veringa et al. [40]	2012	18 months	Pravastatin supplemented with vitamin E after 6 months and homocysteine lowering therapy after other 6 months	93 chronic kidney disease patients	Significant improvement of compliance and distensibility in the common carotid and femoral artery

Shargorodsky et al. [16]	2010	6 months	1,000 mg vitamin C, 400 IU vitamin E, 120 mg co-enzyme Q, 200 mcg selenium	70 patients with multiple cardiovascular risk factors (at least 2)	Significant increase of large and small vessel elasticity

Rasool et al. [41]	2008	2 months	50, 100 and 200 mg/day tocotrienol rich vitamin E	36 healthy men	Improvement of arterial compliance after 100 and 200 mg/day tocotrienol rich vitamin E; NO effect on serum lipids

Kelly et al. [42]	2008	8 hours	Oral dose of 2 g vitamin C	26 healthy human volunteers	No effect on augmentation index and markers of oxidative stress

Plantinga et al. [43]	2007	8 weeks	400 IU vitamin E	30 male with essential hypertension	Beneficial effects on endothelium-dependent vasodilation and arterial stiffness
			1 g vitamin C		

Katayama et al. [44]	2004	2 hours	Single dose 2 g vitamin C before smoking	17 healthy male volunteers	Significant reduction of smoking-induced elevation of brachial-ankle PWV

Mullan et al. [45]	2004	120 minutes	2 g i.v. ascorbic acid	12 healthy men	Pretreatment with ascorbic acid prevented the hyperglycemia induced increase of the central aortic pulse pressure and blood pressure

Mullan et al. [46]	2002	4 weeks	Oral 500 mg ascorbic acid/day	30 patients with type 2 diabetes mellitus	lowered blood pressure, decreased arterial stiffness

Skyrme-Jones et al. [47]	2000	3 months	1,000 IU/day oral vitamin E	41 young diabetic subjects (type 1 diabetes mellitus)	Improvement of endothelial vasodilation; no effect on systemic arterial compliance

**Table 4:** Effect of vitamin B12 supplementation on arterial stiffness in human subjects.

Authors	Year of publication	Follow-up	Dose of vitamin B12	Study population	Results
Van Dijk et al. [48]	2015	2 years	500 *μ*g B12 vitamin	569 hyperhomocystenemic elderly	No effect on PWV or carotid intima-media thickness

Koyama et al. [49]	2010	3 weeks	500 mug methylcobalamin and 15 mg/day folate 3 times weekly	20 patients undergoing hemodialysis	Decreased arterial stiffness
